# Transcriptome analysis reveals cell cycle-related transcripts as key determinants of varietal differences in seed size of *Brassica juncea*

**DOI:** 10.1038/s41598-022-15938-5

**Published:** 2022-07-09

**Authors:** Namrata Dhaka, Rubi Jain, Abhinandan Yadav, Pinky Yadav, Neeraj Kumar, Manoj Kumar Sharma, Rita Sharma

**Affiliations:** 1 Department of Biotechnology, School of Interdisciplinary and Applied Sciences, Central University of Haryana, Mahendergarh, Haryana India; 2 School of Computational and Integrative Sciences, Jawaharlal Nehru University, New Delhi, India; 3 School of Biotechnology, Jawaharlal Nehru University, New Delhi, India; 4 Department of Biological Sciences, Birla Institute of Technology and Science (BITS) Pilani, Pilani Campus, Pilani, Rajasthan India

**Keywords:** Biotechnology, Genetics, Molecular biology, Plant sciences

## Abstract

*Brassica juncea* is an important oilseed crop, widely grown as a source of edible oil. Seed size is a pivotal agricultural trait in oilseed Brassicas. However, the regulatory mechanisms underlying seed size determination are poorly understood. To elucidate the transcriptional dynamics involved in the determination of seed size in *B. juncea*, we performed a comparative transcriptomic analysis using developing seeds of two varieties, small-seeded Early Heera2 (EH2) and bold-seeded Pusajaikisan (PJK), at three distinct stages (15, 30 and 45 days after pollination). We detected 112,550 transcripts, of which 27,186 and 19,522 were differentially expressed in the intra-variety comparisons and inter-variety comparisons, respectively. Functional analysis using pathway, gene ontology, and transcription factor enrichment revealed that cell cycle- and cell division-related transcripts stay upregulated during later stages of seed development in the bold-seeded variety but are downregulated at the same stage in the small-seeded variety, indicating that an extended period of cell proliferation in the later stages increased seed weight in PJK as compared to EH2. Further, k-means clustering and candidate genes-based analyses unravelled candidates for employing in seed size improvement of *B. juncea*. In addition, candidates involved in determining seed coat color, oil content, and other seed traits were also identified.

## Introduction

*Brassica juncea* (Indian mustard) is India’s second-most important source of edible oil and is widely grown in many parts of the world^1^. Seed size is one of the most crucial traits and is one of the principal breeding targets in *B. juncea*^2,3^*.* In dicots, seed development ensues after double fertilization and initiates the growth of the diploid embryo and triploid endosperm, surrounded by the maternal integuments. The growth of the embryo lags behind that of the endosperm. Therefore, seed size is initially determined by the growth of integuments and endosperm^4^. As the seed matures, the embryo fills the whole seed cavity, and replaces the endosperm. The mature seed thus contains a fully developed embryo, endosperm, and seed coat derived from integuments. Therefore, seed size is regulated by coordinated regulation of growth of the diploid zygotic embryo, triploid endosperm, and maternal integuments^5–7^. These stages of seed development have previously been characterized in detail in *B. napus*^8^.

Seed size is a quantitative trait, and several QTLs that regulate seed size have been identified in *Brassica* species^2,9–14^. QTLs of different seed traits frequently show co-localization^15,16^. In *B. juncea*, seed size negatively correlates with seed oil content^17^. Recombinant inbred line (RIL) phenotyping in *B. napus* indicated that seed weight is positively correlated with pod length and protein content but negatively correlated with seed number^18^. Therefore, improvement of seed size through breeding remains challenging. Moreover, due to complex genomic constitution, redundancy due to whole genome triplication, and lack of mutant stocks, the molecular basis of seed size control in *Brassica* species is still unclear^19^. The pieces of evidence from genetic and omic studies and molecular characterization are now starting to accumulate, and their careful assimilation is required to understand the complete picture.

As evidenced in model dicot *Arabidopsis*, seed size is governed by several maternal and non-maternal genetic factors by affecting the final cell number and size in the seed coat and the zygotic tissues^20^. Analysis of the correlation between seed weight and seed size, using genetically diverse inbred lines with contrasting seed weight in *B. napus,* has confirmed that seed diameter, volume, and surface area are highly correlated with seed weight while bulk density is not associated with seed weight^21^. Using high-resolution analysis of seed topology, it has been shown in *B. napus* that the final seed size is determined initially by the seed coat and the endosperm and later by the expansion of the developing embryo^8^. However, the spatial environment provided by the seed coat acts as a limitation for further embryo expansion and acts as a mechanical constraint to prevent further increase in embryo size^8,22^. Further, extensive analysis of topology and lipid imaging has suggested that increasing seed oil content may be challenging; instead, increasing the surface area of seeds may be a reasonable strategy to improve the overall oil content and yield^8^.

**Figure 1:**
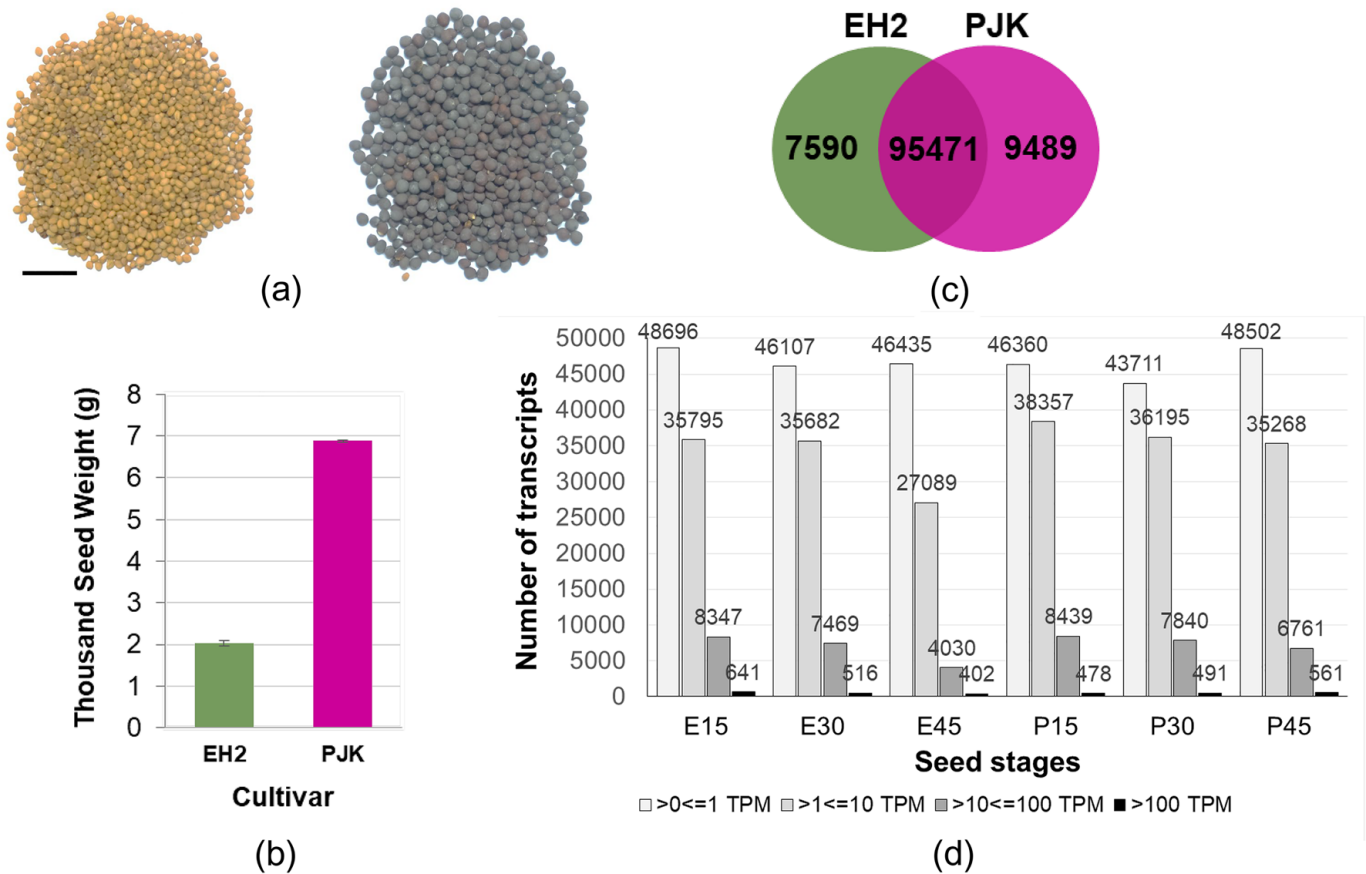
Summary of phenotyping and expression dynamics. (**a**) Picture showing seeds of Early Heera2 (EH2) (yellow colored) and Pusa Jai Kisan (PJK) (brown colored). The scale bar at the bottom corresponds to 1 cm, (**b**) Thousand seed weight (**g**) of EH2 and PJK depicting the contrasting seed weight of both varieties, (**c**) Venn diagram showing the number of transcripts expressed commonly in both varieties, and exclusively in EH2 and PJK, (**d**) Number of transcripts expressed in EH2 and PJK at each of the three stages, according to the level of expression. The legend at the bottom explains the ranges of TPM values.

Transcriptional dynamics of developing seeds have recently been investigated in *B. napus*^23–26^ and *B. rapa*^27^. Here we report transcriptional profiling of developing seeds of two contrasting varieties of *B. juncea*, Early Heera 2 (EH2; small-seeded, yellow seed coat, low oil content) and Pusajaikisan (PJK; bold seeded, brown seed coat, high oil content). EH2 and PJK exhibit contrasting agronomic characters with significant differences in seed size, coat color, and other agronomic traits^2,28^(Fig. 1). In *B. napus,* seed development has previously been classified into three distinct stages viz., 0-18D (days after pollination), 18-40D and 40D-maturity representing the early morphogenic phase marked by the rapid growth of the embryo, middle phase characterized by the accumulation of triacylglycerols, and the desiccation phase, respectively^29^. A similar trajectory of seed development was also confirmed by another study in *B. napus*^8^. Therefore, to investigate the transcriptional dynamics at the landmark stages in *B. juncea*, we performed RNA sequencing from seeds collected at 15D, 30D, and 45D. Hereafter, these stages shall be referred to as early, middle, and late stages, respectively. While this manuscript was in preparation, another study reported transcriptome profiling from EH2 and PJK seeds^30^. However, all three stages used in that study correspond to the first phase of the seed development^30^. By investigating the transcriptional repertoire of early, middle and late seed stages, we demonstrate that the late developmental stages are particularly critical for regulating variety-specific differences in seed size in *B. juncea*.

## Results

### Global expression dynamics during seed development in *B. juncea*

The thousand seed weight of two contrasting varieties of *B. juncea*, EH2 and PJK was measured confirming the extreme variation in seed weight in both varieties, i.e., 2.0 g and 6.9 g, respectively (Fig. 1b). A total of 18 libraries comprising three biological replicates each of early (15D), middle (30D), and late (45D) seed stages from EH2 and PJK were prepared for sequencing. More than 694 billion paired-end reads were obtained. After adapter trimming and quality filtering, > 691 million high-quality reads were retained from 18 libraries (Supplementary Table 1). Read alignment to *B. juncea* reference genome^31^ resulted in 81.0 to 95.4% alignment of all the libraries (Supplementary Table 1). However, one replicate of 30D PJK sample was later removed due to an extremely high percentage of duplicates. Therefore, further analysis was carried out with 17 libraries. All the libraries showed a high correlation coefficient (spearman coefficient > 0.9) between biological replicates (Supplementary Table 2). A total of 137,912 unique transcripts were identified using StringTie. From these, 112,550 transcripts with normalized expression value of transcripts per million (TPM) > 0 were analyzed further. (Supplementary Table 3). Of these, 77,875 were previously annotated in *B. juncea*, while 34,675 were novel transcripts. Further, out of total 112,550 expressed transcripts, 95,471 were expressed in both the varieties, while 7590 and 9489 were detected exclusively in EH2 and PJK, respectively (Fig. 1c). The analysis of transcript abundance showed that ~ 50% of transcripts were detected at TPM > 1 in each of the stages in both the varieties (Fig. 1d). Further, 469 transcripts with TPM > 1 in EH2 but no expression in PJK were referred as EH2-specific while 638 transcripts with TPM > 1 in PJK but no expression in EH2 were identified as PJK-specific (Supplementary Table 4). Some of these transcripts expressed at very high levels with two of the EH2-specific and PJK-specific transcripts having TPM > 100, whereas a set of 41 and 37 EH2- and PJK-specific transcripts, respectively had average TPM > 10. Further, we performed intra- and inter-variety comparisons to identify differentially expressed transcripts at all three stages of seed development.

#### Intra-variety stage-wise differential expression analysis

**Figure 2:**
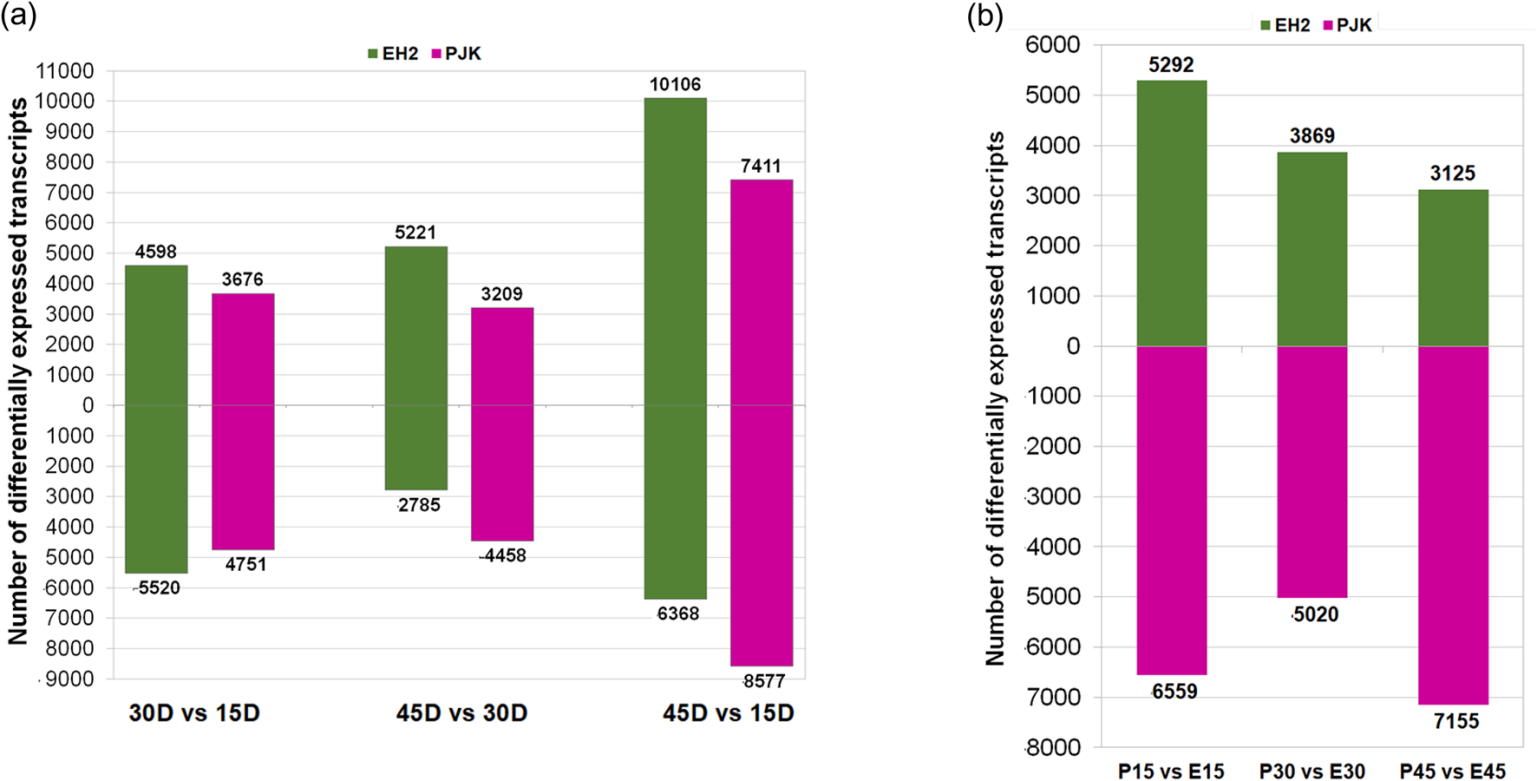
Results of differential expression analysis. (**a**) Differential expression analysis within each variety in three sets, 30D vs. 15D, 45D vs. 30D, 45D vs. 15D. The number of differentially expressed transcripts has been plotted for each comparison in EH2 and PJK. The green bars represent EH2, while the purple bars represent PJK (**b**) Differential expression analysis between the varieties, as compared at each stage, 15D, 30D, and 45D. The green bars represent EH2, while the purple bars represent PJK.

Pairwise differential expression analysis by measuring ≥ two-fold change at q value < 0.05 revealed differentially expressed transcripts at different stages of seed development. A total of 20,514 and 18,901 unique transcripts were differentially expressed in EH2 and PJK, respectively (Supplementary Table 5 and 6). In EH2, 10,118 transcripts were differentially expressed in the middle vs. early stage, of which 4598 and 5520 transcripts were upregulated and downregulated, respectively (Fig. 2a). While in PJK, 8427 transcripts were differentially expressed in the same comparison, and 3676 and 4751 transcripts were upregulated and downregulated, respectively. In the late vs. middle stage, 8006 transcripts were differentially expressed in EH2, of which 5221 and 2785 transcripts were upregulated and downregulated, respectively while 7667 transcripts were differentially expressed in PJK out of which 3209 and 4458 transcripts were upregulated and downregulated, respectively. In late vs. early-stage samples, 16,474 transcripts were differentially expressed in EH2 with 10,106 and 6368 transcripts upregulated and downregulated, respectively. In same comparison, 15,988 transcripts were differentially expressed in PJK, of which 7411 and 8577 transcripts were upregulated and downregulated, respectively. Thus, maximum differential expression was observed in late vs. early stage EH2 and PJK.

#### Inter-variety stage-wise differential expression analysis

In the inter-variety comparison between EH2 and PJK, 19,522 unique transcripts were differentially expressed during seed development (Supplementary Table 7) (Fig. 2b). In the early stage, 11,851 transcripts were differentially expressed in the PJK vs. EH2 comparison, of which 5292 and 6559 transcripts were upregulated and downregulated, respectively. In the middle stage, 8889 transcripts were differentially expressed, of which 3869 and 5020 transcripts were upregulated and downregulated, respectively. In contrast, 10,280 transcripts were differentially expressed in the late stage, of which 3125 and 7155 were upregulated and downregulated, respectively. Thus, the early stage showed maximum differential expression between the varieties, followed by the late and middle stages. Further, in each of the comparisons, the number of transcripts upregulated in PJK was higher than in EH2.

### Functional analysis of stage-wise differentially expressed transcripts in both the varieties

**Figure 3:**
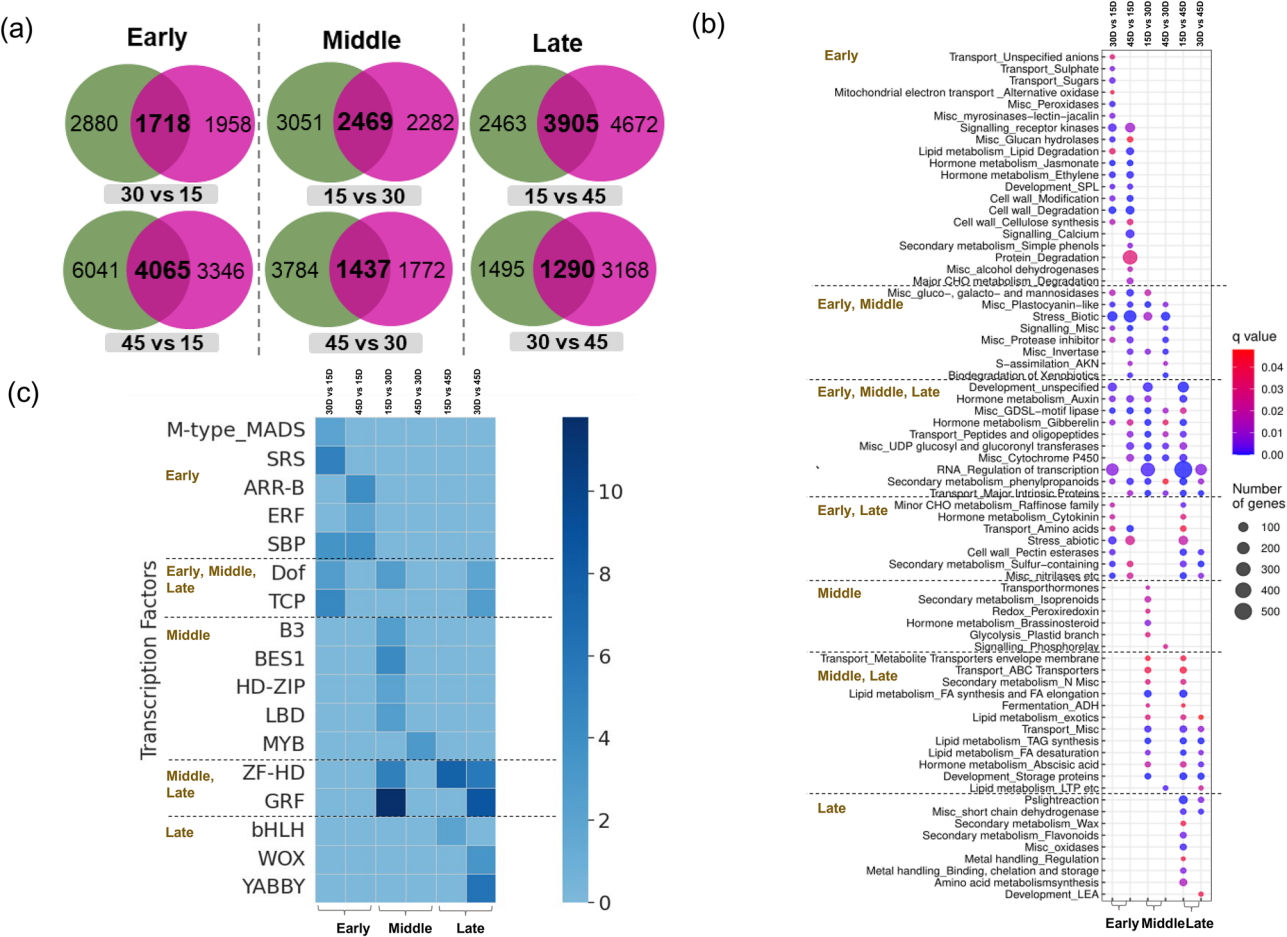
Functional analysis of transcripts upregulated in early (15D), middle (30D), and late (45D) stages of seed development in both EH2 and PJK. (**a**) Venn diagrams showing the number of transcripts upregulated in both EH2 (green) and PJK (purple), as identified through pairwise differential expression analysis between different stages. The number of transcripts shown in bold were commonly upregulated in both varieties in different comparisons and were used for functional analysis (**b**) Pathway mapping results showing the MapMan pathway subcategories enriched in transcripts commonly upregulated at early (30D vs. 15D and 45D vs. 15D comparisons), middle (15D vs. 30D and 45D vs. 30D comparisons), and late (15D vs. 45D and 30D vs. 45D) stages of seed development in both varieties. (**c**) Fold enrichment of transcription factor categories enriched in the transcripts upregulated in early, middle, and late seed development stages in EH2 and PJK.

To dissect the genetic pathways and regulatory modules regulating different stages of development in seeds of *B. juncea*, we separately carried out the functional analysis of transcripts differentially expressed in both varieties at early, middle, and late stages. Firstly, to facilitate functional predictions, we identified the *Arabidopsis* orthologs of all expressed transcripts. A total of 112,550 *Brassica* transcripts mapped to 21,329 *Arabidopsis* genes. Of the 103,061 transcripts expressed in EH2, 96,629 transcripts mapped to 20,880 *Arabidopsis* orthologs, while in PJK, of the 104,960 transcripts, 97,948 mapped to 20,948 *Arabidopsis* counterparts (Supplementary Table 3). Next, using MapMan pathway mapping tool, 73,180 *Brassica* transcripts were mapped to 36 different MapMan pathways and 224 MapMan pathway sub-categories. A total of 7521 transcripts encoding transcription factors could be assigned to 58 TF categories (Supplementary Table 3). Subsequently, to identify the transcripts, which are crucial for regulating early, middle, and late seed development in *B. juncea*, we shortlisted the transcripts exhibiting highest expression at the early, middle, and late stages in both EH2 and PJK (Fig. 3a). The transcripts upregulated in 30 vs. 15D and 45 vs. 15D comparison exhibited highest expression at early stage while those upregulated in 15 vs. 30D and 45 vs. 30D comparison were expressed at highest levels in middle stage. Similarly, transcripts upregulated in 15 vs. 45D and 30 vs. 45D comparison had highest expression at late stages (Fig. 3a). The information about the key genes with known roles in seed traits such as embryo development, seed size, TAG biosynthesis, glucosinolate content, seed coat color, etc. was retrieved from various databases like TAIR (https://www.arabidopsis.org), ARALIP (http://aralip.plantbiology.msu.edu) BRAD (http://brassicadb.cn/#/), and SeedGenes (https://seedgenes.org/index.html) and mapped onto differentially expressed gene sets (Supplementary Table 11). The key findings are elaborated below.

#### Functional analysis of transcripts exhibiting highest expression at the early stage

We observed that 1718 and 4065 transcripts were upregulated at an early stage in EH2 and PJK, compared with the middle and late stages, respectively (Fig. 3a). The key functions assigned to these genes using pathway mapping were cell wall cellulose synthesis, modification, and degradation; signaling receptor kinases; development (SPL); hormone metabolism like jasmonate and ethylene; protein degradation; transport, etc. (Supplementary Table 8) (Fig. 3b). The top GO categories enriched in this gene set were: cell wall, external encapsulating structure, endomembrane system, plasma membrane, apoplast, etc. (Supplementary Table 9). Further, TF fold enrichment at q value < 0.05 showed that transcripts encoding M-type MADS, SRS, ARR-B, ERF, and SBPs TFs were over-represented at the early stage (Supplementary Table 10) (Fig. 3c). Further, a total of 56 transcripts upregulated in the early-stage mapped to 38 *Arabidopsis* genes involved in the regulation of glucosinolate (30.4%), fatty acid elongation, oil content and TAG synthesis (25%), embryo development and endosperm cellularization (16.1%), seed germination and dormancy (16.1%), seed size (5.4%), seed development (3.5%), seed coat color (1.8%), and seed storage (1.8%) (Supplementary Table 11).

#### Functional analysis of transcripts exhibiting highest expression at the middle stage

In the middle stage, 2469 and 1437 transcripts were commonly upregulated in both the varieties compared to early and late stages, respectively (Fig. 3a). They showed enrichment in Mapman pathway sub-categories like brassinosteroid metabolism, hormone transport, signaling, glycolysis, secondary metabolism, etc. (Fig. 3b). GO categories like cell wall, endomembrane system, lipid particle, lipid storage, etc., were also enriched in this dataset (Supplementary Table 9). Further, TFs belonging to B3, BES1, HD-ZIP, LBD, and MYB were overrepresented at the middle stage (Fig. 3c). Candidate gene analysis showed that 168 transcripts upregulated in the middle stage mapped to known candidates, with maximum mapping to fatty acid elongation and synthesis, triacylglycerols (TAG) synthesis, and oil synthesis (47%), followed by embryo development (20.8%), seed coat color, development and mucilage (10.7%), seed germination and dormancy (10.7%), glucosinolate (6.5%), seed development (2.4%), and seed size (1.8%) (Supplementary Table 11).

#### Functional analysis of transcripts exhibiting highest expression at the late stage

In the late stage, 3905 and 1290 transcripts were upregulated as compared to early and middle stages, respectively (Fig. 3a). These were enriched in pathways like light reaction, flavonoid metabolism, development (LEA), etc. (Fig. 3b). GO categories related to photosystem, phenylpropanoid metabolism, secondary metabolites, etc., were enriched in transcripts upregulated at the late stage (Supplementary Table 9). In contrast, TFs like bHLH, WOX, and YABBY were enriched (Fig. 3c). The mapping of 166 transcripts upregulated in late-stage to candidate genes proffered transcripts related to fatty acid elongation and synthesis, oil synthesis and TAG synthesis (56%), seed germination and dormancy (15.1%), embryo development (14.5%), glucosinolate (4.8%), seed coat color, mucilage, and seed coat structure (4.2%), seed development (1.8%), seed maturation (0.6%), seed size (1.8%), and seed storage (0.6%) (Supplementary Table 11).

Apart from the stage-specific pathways, several pathways like regulation of transcription, auxin and gibberellin metabolism, development, cytochrome P450, etc., were enriched at all three stages. Similarly, DOF and TCP TFs were enriched in all the stages of seed development. In contrast, ZF-HD and GRFs were enriched in the middle and late stages.

### Functional analysis of inter-variety differentially expressed transcripts

**Figure 4:**
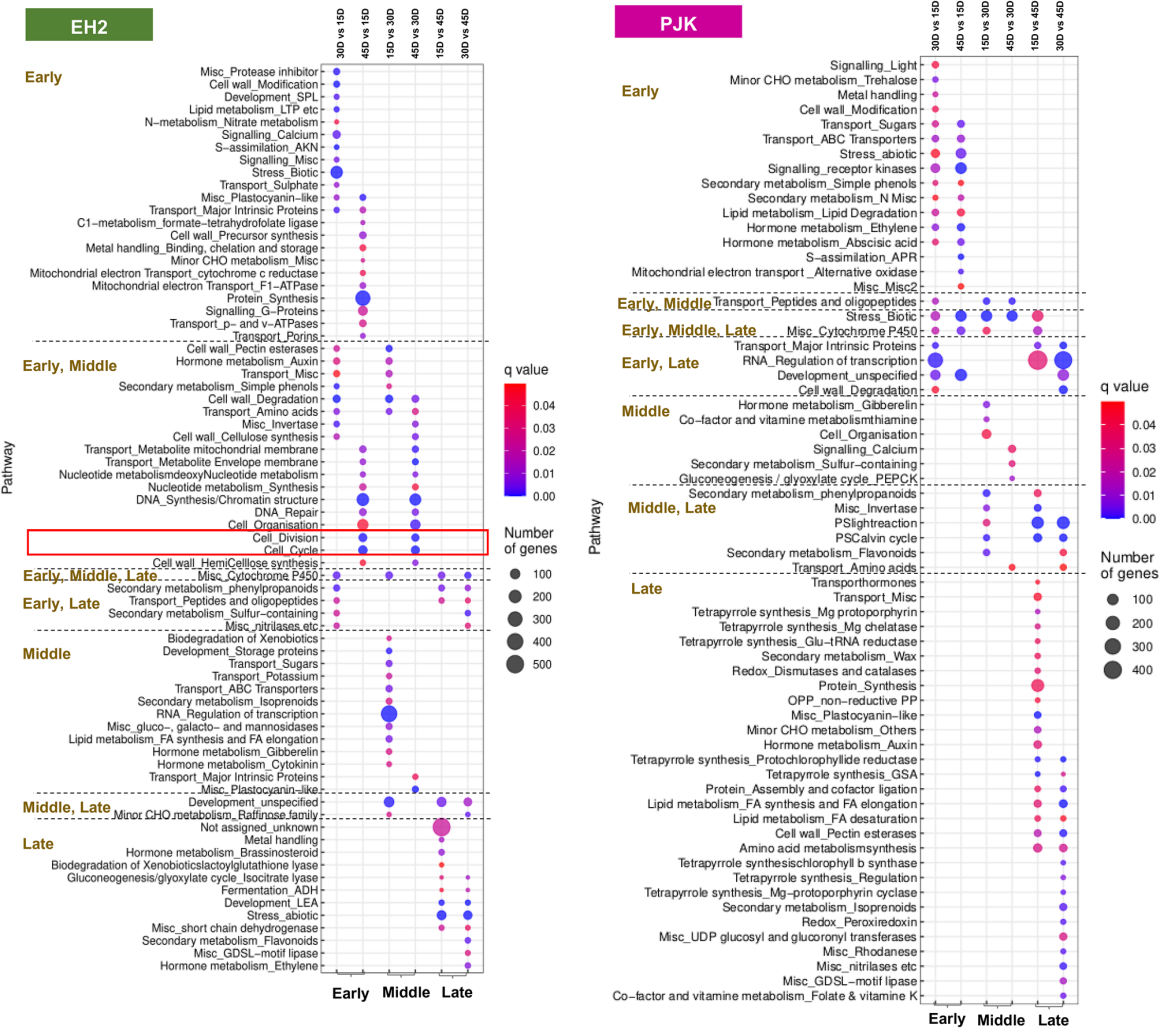
Results of MapMan pathway enrichment for transcripts differentially expressed only in EH2 and those differentially expressed only in PJK. The enriched MapMan pathway subcategories are shown on the left. The *q* value and the number of genes are represented for transcripts upregulated at early, middle, and late stages of seed development in both EH2 and PJK. Transcripts related to cell division and cell cycle are enriched among transcripts upregulated in the early and middle stages in EH2 only.

To identify the critical determinants governing the variety-specific differences concerning seed weight, we first analyzed the transcripts upregulated at each stage in a variety-specific manner (Fig. 4) (Supplementary Tables 5 and 6). Among the transcripts upregulated in EH2 only, the topmost enriched pathways at the early stage were biotic stress, protein synthesis, S-assimilation, cell wall modification, protease inhibitor, etc. Those involved in DNA synthesis, cell cycle, cell division, cell wall degradation, auxin, etc. were enriched at both early and middle stages, while cytochrome P450 genes were enriched at early, middle, and late-stages. Similarly, transcripts involved in secondary metabolism, peptide transport, etc. were enriched at the early and late stage. Those involved in regulation of transcription, development—storage proteins, transport—sugars, ATP-binding cassette (ABC) transporters, lipid metabolism, plastocyanin, etc. were enriched at the middle stage while transcripts regulating development and minor carbohydrates metabolism were enriched in middle and late-stage. Further, transcripts regulating abiotic stress, development—LEA, flavonoid metabolism, brassinosteroid, and ethylene metabolism, etc. were enriched at the late-stage. The enriched GO categories also reflected the same pattern with cell cycle and DNA replication among the top categories in the early and middle stages (Supplementary Table 9). On the contrary, among the transcripts upregulated in PJK only, the topmost enriched pathways belonged to categories S- assimilation, signaling receptor kinases, ethylene, and abscisic acid (ABA) metabolism, mitochondrial electron transport, transport sugars, etc. at the early stage while those involved in peptide and protein transport were enriched at early and middle stages. Transcripts associated with biotic stress and cytochrome P450 were enriched in early, middle, and late-stages, while those associated with transport—major intrinsic proteins, cell wall degradation, regulation of transcription, development were enriched at early and late stages. Similarly, gibberellin metabolism, gluconeogenesis, calcium signaling, etc.-associated transcripts were enriched at the middle stage and those associated with light reaction, Calvin cycle, flavonoid metabolism, etc. were enriched at the middle and late-stages. The transcripts involved in protein synthesis, lipid metabolism, tetrapyrrole synthesis, cell wall pectin esterases and secondary metabolism were enriched at late-stage.

**Figure 5:**
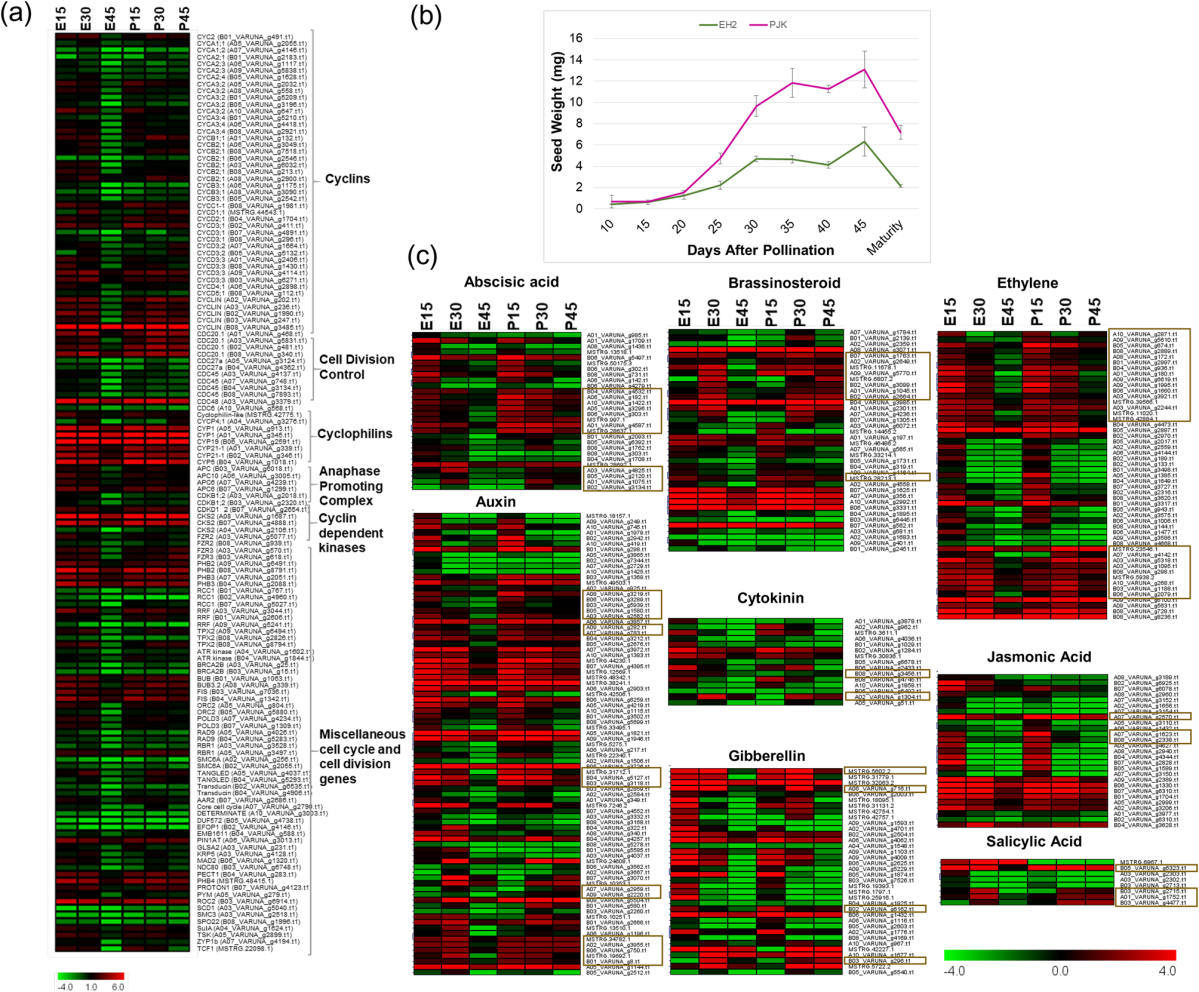
Identification of candidate genes responsible for variety-specific differences in cell proliferation during the late stage of seed development. (**a**) Heatmap showing expression of cell cycle and cell division related transcripts upregulated in early and middle stage only in EH2. Most of the transcripts exhibit a decrease in expression in E45 but not in P45 (**b**). Analysis of seed weight (mg) in EH2 and PJK from 10 days after pollination till maturity. Fresh weight is shown from 10 to 45D, and dry weight is shown at maturity. An average weight of twenty seeds was used, and three replicates were used. Standard deviation is also shown. EH2 and PJK exhibit similar seed weight till 15D, and maximum seed weight difference can be observed 30D to 45D. (**c**) Heatmaps showing expression patterns of transcripts mapped to eight phytohormones based on MapMan pathway mapping. The transcripts upregulated in the early and middle stages only in EH2 are shown. The boxes demarcate the transcripts, which show a decrease in expression in E45 but not in P45, indicating candidates for phytohormones transcripts responsible for continued cell proliferation in PJK.

Interestingly, cell division and cycle-related pathways were enriched among the transcripts upregulated at both early and middle stages in EH2 only, while their expression goes down in the late stage of seed development (Fig. 4). Since cell proliferation is a significant determinant of seed size regulation, this observation indicates that the cell division and cell cycle transcripts might play a key role in regulating the seed size difference among these varieties. Therefore, we shortlisted the transcripts belonging to cell division and cell cycle pathway categories which were uniquely upregulated at 15D and 30D compared to 45D in EH2 only (Supplementary Table 12). We obtained a total of 136 transcripts. While these transcripts showed a reduction in expression at the 45D stage in EH2, most of these did not show the reduction in the 45D stage in PJK (Fig. 5a). This expression pattern correlates with the differences observed in seed weight in these varieties, as the continuous assessment of seed weight from 10D to the mature stage showed that the seed weight in both the varieties does not exhibit much difference in the early stages, and the maximum seed weight difference is observed at the later stage of seed development, i.e., from 30 to 45D (Fig. 5b). Identification of the orthologous genes from *Arabidopsis* revealed that 45 of these transcripts were cyclins, 12 were *cell division control (CDC)* transcripts, 7 were *Cyclophilins*, 4 were *Anaphase Promoting Complex (APC)* transcripts, 3 were *Cyclin-dependent kinases (CDK)*, and the rest were miscellaneous cell cycle-related transcripts like *core cell cycle*, *retinoblastoma-related (RBR)* family, *pin-formed (PIN)* protein family, *Ataxia-telangectasia* and *Rad3 related (ATR) kinase,* etc. (Supplementary Table 12).

To further identify the candidates that may be important in regulating the cell cycle and cell division-related transcripts identified above, we shortlisted the phytohormones-related transcripts specifically upregulated at 15D and 30D compared to 45D in EH2 only. MapMan pathway mapping results showed that a total of 286 transcripts in this category mapped to phytohormones, corresponding to ABA (28), auxin (83), brassinosteroid (38), cytokinin (15), ethylene (51), gibberellin (36), jasmonate (27), and salicylic acid (8) biosynthesis/signaling (Fig. 5c, Supplementary Table 13).

**Figure 6:**
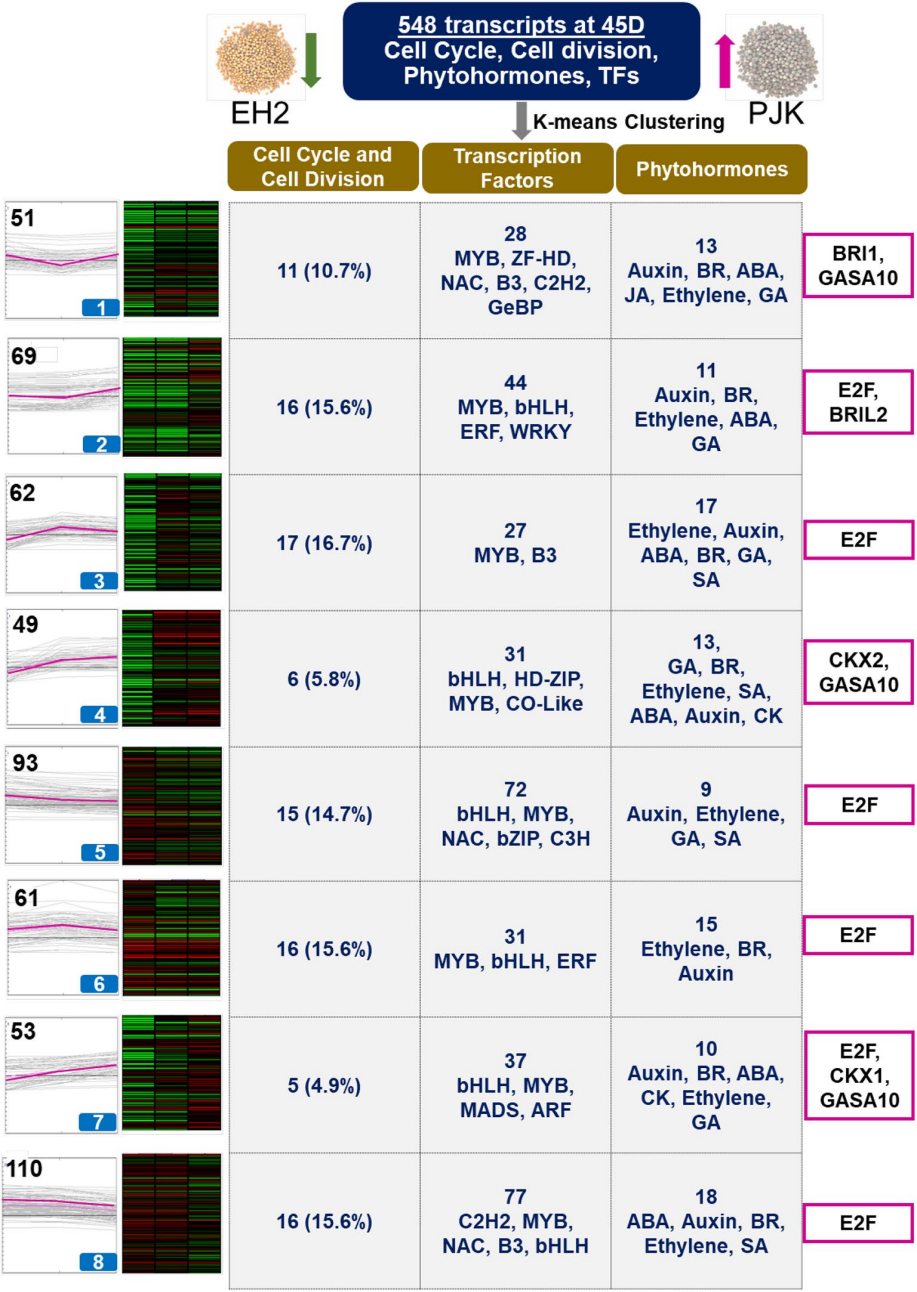
Clustering analysis of transcripts upregulated in the late seed development stage in PJK compared to EH2 (P45D vs. E45D). 548 transcripts belonging to pathway categories cell cycle, cell division, phytohormones, and transcription factors were used for k-means clustering in MeV using the log-transformed TPM values. 548 transcripts clustered into eight groups, as shown on the left. The number of transcripts belonging to each category (cell cycle and cell division, transcription factors, phytohormones) is shown. The percentage of cell cycle-related genes out of total mapping to each cluster is also shown, depicting that maximum cell cycle-related transcripts clustered in group 3 and lowest in group 5. The top categories of TFs and phytohormones are also shown. The purple boxes on the right side depict the most crucial candidates for the regulation of cell proliferation in developing seeds in each group.

To further dissect the cell cycle and division-related transcripts governing variety-specific differences in seed weight, we investigated the transcripts upregulated at the late stage in PJK compared to EH2. A total of 10,280 transcripts were differentially expressed in PJK vs. EH2 at 45D, of which 7155 transcripts were upregulated in PJK (Fig. 2b). Of these, 102 transcripts were related to cell cycle and cell division, as shown by pathway mapping (Supplementary Table 14). Further, we identified 446 phytohormones and TF-related transcripts upregulated at 45D in PJK vs. EH2. (Supplementary Table 14). Of these 548 transcripts, 11 were expressed in a PJK-specific manner (A01_VARUNA_g4163.t1, A03_VARUNA_g1726.t1, A03_VARUNA_g820.t1, B02_VARUNA_g6466.t1, B04_VARUNA_g3041.t1, B05_VARUNA_g1330.t1, B05_VARUNA_g1773.t1, B07_VARUNA_g6715.t1, MSTRG.19891.1, MSTRG.3848.1, MSTRG.42813.2). Next, using the 548 transcripts, we carried out k-means clustering using MeV to identify coexpressed transcripts to narrow down the regulatory modules. After clustering, these 548 transcripts were grouped into eight different groups (Fig. 6). Out of all the cell cycle and cell division transcripts, maximum (17) grouped into cluster 3, which showed the prevalence of MYB and B3 TFs and transcripts mapping to all six phytohormones, including auxin, ABA, brassinosteroid, ethylene, gibberellin, and salicylic acid. Three clusters, 2, 6, and 8, harbored 16 cell cycle and cell division transcripts. Clusters 5, 1, and 4 harbored 15, 11, and 6 transcripts, respectively, while the smallest cluster 7 harbored five transcripts related to cell cycle and division. MYB TFs were the top TFs present in all the clusters. Among phytohormones, auxin was a top hormone in most of the clusters. Based on the insights from the literature, some of the crucial candidates like *E2F, BRI1(BRASSINOSTEROID INSENSITIVE1), BRIL2, GASA10 (GIBBERELLIC ACID STIMULATED ARABIDOPSIS 10), CKX1 (CYTOKININ DEHYDROGENASE 1),* and *CKX2 (CYTOKININ DEHYDROGENASE 2)* were predicted as key regulators among these clusters.

### Orthology-based identification of candidate genes for regulating seed size, coat color and oil content in *B. juncea*

**Figure 7:**
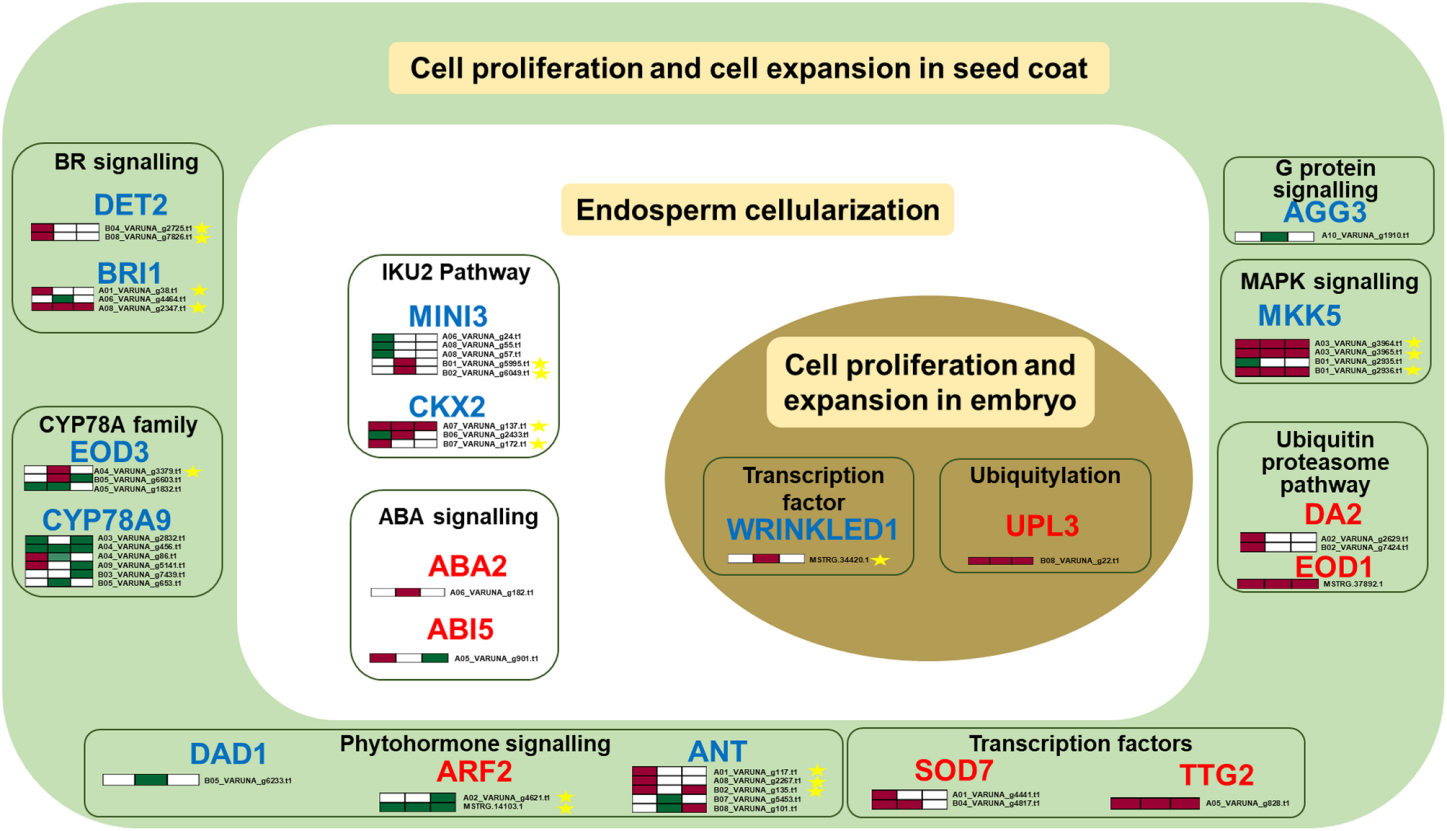
Identification of candidate genes responsible for variety-specific differences in seed weight in *Brassica juncea*. Candidate genes regulating seed size/weight were identified from the literature. Seed size is mainly regulated by genes regulating cell proliferation or expansion in the seed coat, endosperm, or embryo. Those genes which showed differential expression between PJK and EH2 are shown here. Positive regulators are shown in blue and negative regulators are shown in red. Heatmaps are shown for orthologous transcripts in *B. juncea* transcriptome, only for the transcripts differentially expressed at early, middle, or late stages of seed development. Color coding for heatmap: white—not differentially expressed, green—upregulated in EH2, purple—upregulated in PJK. Yellow stars mark the transcripts that are good candidates responsible for variety-specific seed size differences based on our transcriptome analysis.

We identified candidate genes regulating seed weight from the literature to further shortlist the candidates regulating variety-specific differences in seed size. Based on the knowledge from *Arabidopsis*, several pathways, including transcriptional regulators, phytohormones, signaling, and other players, are well elucidated for their roles in governing seed size^32^. We identified such candidate genes and observed that 79 transcripts, orthologous to 29 *Arabidopsis* genes, were differentially expressed between EH2 and PJK (Supplementary Table 15). This comparison unravelled that orthologous transcripts of at least eight positive regulators (*BRI1, DET2 (DE-ETIOLATED-2), EOD3 (ENHANCER3 OF DA1), MINI3, CKX2, WRI1 (WRINKLED1), ANT (AINTEGUMENTA*)*,* and *MKK5 (MITOGEN-ACTIVATED PROTEIN KINASE KINASE5*) were upregulated in PJK (bold-seeded) and downregulated in EH2 (small-seeded), and those orthologous to one negative regulator (*ARF2; AUXIN RESPONSE FACTOR2*) was upregulated in EH2 and downregulated in PJK (Fig. 7).

Further, we also leveraged the information available in TAIR, ARALIP, SEEDGENES, and BRAD databases for *Arabidopsis* gene functions of genes, to identify candidate genes regulating seed coat color and oil content (Supplementary Table 16). EH2 is a yellow-seeded variety with ~ 36.9% oil content, while PJK is a brown seeded variety with ~ 41.1% oil content^28^ (Fig. 1). Orthologs of at least 14 seed coat color-related and 17 oil content-related *Arabidopsis* genes showed differential expression in EH2 and PJK indicating their potential involvement in regulating contrasting phenotypes (Table 1). We also analysed the highest expressing transcripts in each of the stages, in both the varieties, to determine if any of these are interesting candidates based on inter-varietal differential expression. For this, we shortlisted the top 100 expressing transcripts in 15D, 30D, and 45D stages of both EH2 and PJK. Among these, we obtained 11, 7, 37 highest expressing transcripts in 15D, 30D, and 45D respectively which showed significant upregulation in EH2 as compared to PJK at the respective stages, and 12, 18, and 22 transcripts that were highly expressed in 15D, 30D, and 45D in PJK and also significantly upregulated in PJK as compared to EH2 at the same stage (Supplementary Table 17). Based on overlaps with the pathway mapping and candidate genes results, we observed an interesting finding that of the 37 of these highest expressing transcripts upregulated in 45D in EH2, 10 were orthologs of *Arabidopsis* oleosin genes involved in oil biosynthesis (Supplementary Table 16 and 17), indicating that these may be good candidates for further dissecting the differences in lipid metabolism in EH2 and PJK.

**Table 1:** Transcripts shortlisted as candidates for seed coat color and oil content based on differential expression between PJK and EH2 and known roles in *Arabidopsis* (* represents ‘not differentially expressed’).

S. No	*Bju* Transcript ID	Fold change			AT ID	Candidate genes	Function
		P15 vs. E15	P30 vs. E30	P45 vs. E45			
**Seed coat color**							
1	B01_VARUNA_g1437.t1	− 9.7	− 11.1	− 9.4	AT1G61720	*BAN*	Negative regulator of flavonoid biosynthesis that prevents pigment accumulation^82^
2	A01_VARUNA_g2946.t1	− 8.6	− 9.1	− 6.8			
3	A01_VARUNA_g3406.t1	− 8.1	− 9.4	− 9.2			
4	B05_VARUNA_g3917.t1	*	*	− 1.7	AT1G53500	*MUM4*	Mucilage synthesis or secretion in the seed coat^83^
5	A05_VARUNA_g1704.t1	1.8	*	*			
6	A06_VARUNA_g104.t1	1.4	*	*			
7	A06_VARUNA_g613.t1	6.2	4.7	*	AT1G09540	*MYB61*	Mutant seeds lack mucilage extrusion and deposition^84^
8	B06_VARUNA_g5004.t1	*	2.4	− 1.9	AT1G34790	*TT1*	Mutant seeds display yellow color due to lack of tannin pigments^85^
9	A09_VARUNA_g2999.t1	*	1.1	*			
10	A02_VARUNA_g4188.t1	− 3.6	2.4	*	AT5G48100	*TT10*	Mutants exhibit delay in seed coat browning due to the oxidation of flavonoids^45^
11	A06_VARUNA_g3597.t1	*	2.5	1.4			
12	B05_VARUNA_g1350.t1	− 10.8	− 10.3	− 7.0	AT3G59030	*TT12*	Recessive mutant seeds show a pale brown color with a reduction of proanthocyanidins deposition in endothelium^86^
13	A07_VARUNA_g2393.t1	− 1.9	− 3.8	− 4.8			
14	MSTRG.4757.2	− 1.2	*	*	AT5G23260	*TT16*	Accumulates proanthocyanidin in the endothelium^87^
15	B08_VARUNA_g1307.t1	2.1	*	*			
16	B02_VARUNA_g2630.t1	− 6.0	− 5.3	− 3.2	AT5G17220	*TT19*	Mutants have anthocyanins but do not have the brown pigments^88^
17	A02_VARUNA_g716.t1	− 4.1	− 7.4	− 4.9			
18	B01_VARUNA_g4359.t1	*	*	− 1.0	AT5G35550	*TT2*	Accumulate proanthocyanidins in the endothelium during early seed development^89^
19	B06_VARUNA_g1751.t1	− 10.6	− 10.9	− 10.1	AT5G42800	*TT3*	Mutants show yellow seed coat^90^
20	A10_VARUNA_g2403.t1	− 3.4	− 5.7	− 3.5	AT5G13930	*TT4*	Involved in the biosynthesis of flavonoids^91^
21	A03_VARUNA_g575.t1	− 3.4	− 5.6	− 2.2			
22	A02_VARUNA_g536.t1	− 3.4	− 7.4	− 3.5			
23	B02_VARUNA_g2406.t1	*	− 2.3	− 3.9			
24	B08_VARUNA_g4097.t1	*	− 1.4	− 3.3			
25	A02_VARUNA_g4226.t1	6.7	*	− 2.6			
26	B03_VARUNA_g623.t1	*	− 2.4	− 1.9			
27	B06_VARUNA_g557.t1	3.6	*	*			
28	MSTRG.49249.1	*	− 1.7	*	AT5G07990	*TT7*	Mutants show altered levels and patterns of proanthocyanidin deposition^92^
29	B03_VARUNA_g5220.t1	− 3.5	*	− 1.7	AT4G09820	*TT8*	Regulates phenylpropanoid synthesis in seed coat^71^
30	A05_VARUNA_g828.t1	− 2.8	− 1.6	− 3.1	AT2G37260	*TTG2*	Control the production of proanthocyanidin in the inner testa layer^46^
**TAG biosynthesis**							
1	B07_VARUNA_g4392.t1	*	1.8	*	AT3G24650	*ABI3*	Overexpression causes enhanced lipid content^93^
2	MSTRG.7408.1	*	1.4	1.5			
3	B03_VARUNA_g2458.t1	1.1	2.1	*	AT2G40220	*ABI4*	Mutants showed TAG accumulation and reduced expression of *DGAT1*^94^
4	A05_VARUNA_g590.t1	*	4.1	*			
5	B05_VARUNA_g6109.t1	*	1.3	*			
6	MSTRG.39372.1	*	3.2	*	AT3G44460	*bZIP67/DPBF2*	Overexpression inhibits the expression of *FAD3*, which results in increased 18:1 and decreased 18:3 levels in seeds^95^
7	A06_VARUNA_g2114.t1	*	1.3	*			
8	A09_VARUNA_g1148.t1	*	*	− 2.8	AT2G19450	*DGAT1*	Seed-specific overexpression increases oil content from 11 to 28%^96^
9	A03_VARUNA_g4555.t1	− 1.6	*	*	AT3G51520	*DGAT2*	Mutants show defective TAG biosynthesis^97^
10	A05_VARUNA_g3512.t1	− 4.5	− 4.8	− 4.4	AT3G12120	*FAD2*	Mutants are deficient in oleate desaturase^98^
11	A05_VARUNA_g1444.t1	2.3	1.3	− 1.9	AT2G29980	*FAD3*	Loss of function mutants show increased activity of C18:2 desaturase and drastically lower C18:3 content in the endoplasmic reticulum^99^
12	B03_VARUNA_g1718.t1	1.8	*	*			
13	B05_VARUNA_g4686.t1	1.7	*	1.0			
14	B04_VARUNA_g1017.t1	*	2.2	1.1			
15	A04_VARUNA_g2237.t1	*	1.1	1.7			
16	A03_VARUNA_g1569.t1	*	*	3.6			
17	A02_VARUNA_g3954.t1	4.7	*	*	AT3G26790	*FUS3*	Overexpression increases oil content^100^
18	B08_VARUNA_g2257.t1	4.4	*	*			
19	B06_VARUNA_g751.t1	3.5	1.2	*			
20	A06_VARUNA_g3829.t1	2.8	*	*			
21	A02_VARUNA_g1642.t1	*	− 2.5	6.9	AT5G50700	*HSD1,MID1*	Ectopic expression deprived the accumulation of fatty acids in seed oil bodies^101^
22	B03_VARUNA_g3285.t1	*	2.5	2.9			
23	B02_VARUNA_g5986.t1	*	*	5.7			
24	A03_VARUNA_g2629.t1	*	*	5.0			
25	A04_VARUNA_g2274.t1	*	− 1.0	*	AT2G30470	*HSI2/VAL1*	Acts as a negative regulator for oil content and seed storage proteins^102^
26	B04_VARUNA_g982.t1	1.9	*	*			
27	A03_VARUNA_g1603.t1	1.3	*	*			
28	B03_VARUNA_g1756.t1	*	1.5	*			
29	MSTRG.15960.1	*	*	− 2.8	AT1G21970	*LEC1*	Loss-of-function causes defects in storage protein and lipid accumulation^103^
30	A09_VARUNA_g4072.t1	6.2	*	− 1.4			
31	B06_VARUNA_g3502.t1	6.8	*	− 1.2			
32	MSTRG.45383.2	1.9	*	*			
33	A09_VARUNA_g3595.t1	*	*	− 1.7	AT1G28300	*LEC2*	Ectopic expression causes the accumulation of seed storage proteins and lipid oil bodies in vegetative and reproductive organs^104^
34	B07_VARUNA_g5614.t1	5.4	1.1	1.6	AT4G25140	*OLE1*	Deficiency in OLE1 causes a decrease in total lipid content^105^
35	A03_VARUNA_g5249.t1	*	3.8	3.3			
36	A01_VARUNA_g1620.t1	*	3.2	2.9			
37	B02_VARUNA_g1185.t1	*	1.9	2.7			
38	A08_VARUNA_g2121.t1	*	1.6	1.6			
39	B07_VARUNA_g5614.t1	5.4	1.1	1.6			
40	B04_VARUNA_g2151.t1	*	4.5	2.3	AT5G40420	*OLE2*	Suppression in *OLE2* causes enlarged oilbodies^106^
41	A04_VARUNA_g1362.t1	*	4.3	3.5			
42	A07_VARUNA_g2032.t1	*	1.8	2.5			
43	A03_VARUNA_g3061.t1	*	2.7	1.6	AT3G01570	*OLE4*	Negatively regulate the size of oil body^107^
44	B01_VARUNA_g2620.t1	*	*	1.4			
45	B01_VARUNA_g4359.t1	*	*	− 1.0	AT5G35550	*TT2*	Mutant seeds show enhanced seed FA accumulation^108^
46	B03_VARUNA_g5220.t1	− 3.5	*	− 1.7	AT4G09820	*TT8*	Double mutants of *BnTT8* produce seeds with elevated seed oil content^71^

## Discussion

### Identification of conserved hallmarks regulating temporal stages of seed development in *Brassica juncea*

We utilized two approaches to dissect the global transcriptional dynamics during the early, middle, and late stages of seed development in *B. juncea*. In the first approach, we identified commonly upregulated transcripts in both the varieties at different stages of development, and dissected functional categories and candidates important for governing early, middle, and late seed development in *B. juncea*. Several key transcripts known as hallmarks of embryo development and endosperm cellularization, seed maturation and filling, and desiccation and dormancy were detected at early, middle and late stages of seed development, respectively.

The functional categories enriched among transcripts upregulated at an early stage highlighted cell wall-related transcripts. It is well evidenced that cell wall loosening and weakening are crucial for the growth of developing embryos^33^. Several MADS and MYB TFs, enriched in the early stage in our data, are also known to regulate embryo development in *Arabidopsis*^34^*.* One of the most well-known candidates was *MINISEED3 (MINI3)*, which encodes a WRKY10 transcriptional activator and acts as a pivotal regulator of embryo development and cell size by regulating cell division and elongation^35^. It is also recently shown in *B. napus* as a potential regulator of CK responses in developing endosperm^36^. Some of the other important candidates were homologs of *AMINO ACID PERMEASES1* (*AAP1), ARABIDOPSIS FORMIN HOMOLOGY5 (AtFH5), (RETARDED GROWTH OF EMBRYO1 (RGE1),* etc. *AAP1* imports amino acids in the embryos and affects the content of carbon, nitrogen, and seed storage compounds^37^. *AtFH5* codes for a formin protein, crucial for maintaining actin organization and cytokinesis, and is important for normal endosperm cellularization^38^. *RGE1* codes for a bHLH TF, which regulates embryo development^39^.

The pathway categories enriched in the middle stage also conformed to the physiological changes during the middle stage of seed development. For example, brassinosteroids (BRs) regulate embryo and endosperm development^40^. B3 TFs, enriched in the middle stage, consist of several crucial regulators of seed storage accumulation like *ABSCISIC ACID INSENSITIVE3 (ABI3), FUSCA3 (FUS3), LEAFY COTYLEDON2 (LEC2)*, etc. These are the master regulators of the seed maturation programs in dicot seed development^41^. Several crucial candidates upregulated in the middle stage were orthologs of genes regulating oil content and quality. For instance, in *B. napus*, simultaneous silencing of *FATTY ACID DESATURASE2 (FAD2)* and *FATTY ACID ELONGASE1 (FAE1)* has been shown to reduce the content of polyunsaturated fatty acids (PUFA) and erucic acid and increase the content of oleic acid and proteins in the oil^42,43^. *FUS3* expresses during embryo maturation and is a significant regulator of seed filling during seed maturation^44^. Seed coat-related transcripts were also prominent in transcripts upregulated at the middle stage, for example, *TRANSPARENT TESTA4 (TT4), TT10, TT16, TRANSPARENT TESTA GLABRA2 (TTG2)*, etc. *TT10* codes for a laccase-like enzyme involved in the polymerization of flavonoids and regulates the browning of the seed coat^45^. *TTG2* encodes a WRKY TF that controls the deposition of tannins in the seed coat^46^.

The late-stage-upregulated transcripts were mainly related to seed dormancy and desiccation-related functions. For example, LEA proteins are crucial for desiccation and seed longevity in mature seeds^47^. bHLH TFs are known to participate in the regulation of *DELAY OF GERMINATION1 (DOG1)*, which regulates seed dormancy^48^. Eight transcripts orthologous to *DOG1* showed upregulation among the dormancy-related transcripts at the late stage. *DOG1* is a significant regulator of seed dormancy and longevity, as shown in several species like *Arabidopsis*, wheat, lettuce, etc. It regulates dormancy in response to various endogenous and environmental factors by affecting multiple germination-related processes like activation of storage compounds, weakening seed coats, cell elongation, etc.^49^. These observations not only confirm that we could successfully capture the transcriptional repertoire regulating landmarks stages of seed development in *B. juncea* but also highlights high conservation in the key regulators modules regulating seed development in *Arabidopsis* and *Brassica*.

### An extended period of cell proliferation is a possible cause of increased seed size in the bold-seeded variety

In the second approach, we used the transcripts upregulated explicitly in each of the varieties at different stages, and identified the crucial candidates governing variety-specific differences in seed size. We identified cell cycle and cell division-related transcripts as significant determinants of seed size. These were orthologous to genes encoding cyclins and CDKs, the regulatory and catalytic subunits of the protein kinases that regulate the cell cycle progression. For example, CDCs are crucial for DNA replication initiation while APCs are essential for cell cycle phase transition^50^. The decrease in expression of these transcripts in late-stage in EH2, but not PJK, and the upregulation of several cell cycle-related transcripts in the late stage of PJK compared to EH2 indicates that cell division and cell cycle-related genes stay upregulated during later stages in PJK. At the same time, they are downregulated in EH2, suggesting that an extended period of cell proliferation is likely responsible for increased seed weight in PJK compared to EH2.

Interestingly, a similar finding has been recently made in *B. rapa*^27^. Based on the differential expression analysis between the seeds of high and low seed size cultivars, Niu et al. 2020^27^ concluded that cell division and cell cycle are the significant determinants of seed size in *B. rapa*. They also suggested that the duration of cell cycle-specific gene expression at different seed development stages may be instrumental in determining seed size differences^27^. Further, Li et al. 2015^21^ have also demonstrated that seed weight exhibits a higher correlation with cell number rather than the cell size of cotyledons and seed coats. These observations also hold up in *B. napus*^18^, indicating that cell number rather than cell size is the primary determinant of seed weight in *Brassica*. In another dicot, chickpea, an extended period of cell proliferation has been determined to contribute to cultivar-specific differences in seed weight^51^.

Since phytohormones are crucial regulators of the cell cycle, and auxins and cytokinins especially are classically known as positive regulators of cell proliferation, we also identified the phytohormone-related transcripts which may be responsible for regulating the cell cycle-related transcription^52,53^. Coexpression analysis suggested that TFs like E2Fs and phytohormones including BRs, CKS, GAs and auxins contribute to differential levels of cell cycle and cell division in developing seeds. E2Fs are well known for regulating the cell cycle during seed development^50^ and were represented in 6 of the 8 clusters. E2F and B3 TFs were also found to coexpress with cell cycle genes in *B. napus* and were identified as possible hub genes for regulating cell proliferation in developing seeds^27^. BRs regulate cell division positively, and *BRI1* mutants exhibit a decrease in seed size. Hence, *BRI1* is a valuable candidate for transcripts clustered in group 1. GASA genes express in response to GAs, and *GASA10* is previously known to regulate cell elongation positively^54^. Therefore, *GASA10* may also be a useful candidate for regulating cell cycle-related transcripts. CKXs are cytokinin dehydrogenases regulating cell proliferation^55^ and are thus, other potential candidates in the clusters. In *B. napus*, the cytokinin oxidase/dehydrogenase (CKX) gene family has been predicted to be a good candidate for manipulating seed size based on the transcriptome analysis of developing seeds and other tissues^56^.

Furthermore, candidate gene-based analysis identified orthologs of at least eight genes (*BRI1, DET2, EOD3 MINI3, CKX2, WRI1, ANT, MKK5*) which may be involved in regulating seed size differences in EH2 and PJK (Fig. 7). Among these, *BRI1, DET2, EOD3, ANT*, and *MKK5* mainly regulate cell proliferation in the seed coat. *MINI3* and *CKX2* mainly control cell division in the endosperm, and *WRI1* is primarily involved in regulating cell division in embryos. On the other hand, the negative regulator *ARF2* mainly restricts cell division in the seed coat^57^. *BRI1* and *DET2* are related to BR signaling-mediated control of seed size. *BRI1* mutants are BR insensitive and defective in BR reception. *DET2* regulates cell size and cell number in embryos and cell size in integuments positively^58^. In our data, both orthologs of *DET* exhibited upregulation in PJK in the early stage. *EOD3* codes for CytochromeP450/CYP78A6 and acts as an enhancer of *DA1-1*. It positively regulates the cell size of the integument ^59^. *ANT* codes for an AP2 (APETALA2) TF and promotes cell proliferation in the integuments^60^. It has also been shown to act downstream of TF MEE45 (*MATERNAL EFFECT EMBRYO ARREST45)*. *ANT* positively regulates the auxin signaling and promotes cell proliferation in integuments and possibly embryo^61^. Only two of the *Brassica* orthologs of *ANT* showed upregulation in EH2 in the middle stage. Two orthologs showed upregulation in PJK in the late stage, and three orthologs showed upregulation in the early stage (Supplementary Table 15). *MKK5* is a positive maternal regulator of seed size^62^, and three out of four orthologous transcripts showed upregulation in PJK in all three stages of seed development. *MINI3*, a WRKY TF, expressed in both endosperm and embryo, is a positive regulator of seed size. It regulates endosperm size and thus seed size. Mutants of *MINI3* exhibit reduced seed size^63,64^. Two orthologs of *MINI3* exhibited upregulation in PJK in the early stage, indicating its possible involvement in seed size regulation. *CKX2* degrades cytokinin and thus reduces cytokinin signaling in the micropylar endosperm and controls endosperm size via the IKU2 pathway. Reduction of cytokinin activity through the IKU pathway is required for the active growth of endosperm. Hence, *CKX2* is also a positive regulator of endosperm and seed size^65^. Of the three orthologs showing differential expression in our data, one showed upregulation in all three stages of seed development in PJK (Supplementary Table 15).

Further, overexpression of *Arabidopsis WRI1* enhances seed weight in *Camelina sativa*, primarily through cell expansion in embryos^66^. We observed upregulation of *WRI1* ortholog in the middle stage in PJK. *ARF2* is a negative regulator of seed size, repressing cell proliferation in the seed coat through mediating auxin signaling^67^, and was upregulated in EH2 and downregulated in PJK at all three stages of seed development (Supplementary Table 15). Thus, these transcripts are good candidates for investigating variety-specific differences in seed size in *B. juncea*. *ARF2* and *ANT* are the most interesting, as *ARF2* represses *ANT* by binding to its promoter, and *ANT* positively regulates cell proliferation^68,69^. In our study, *ARF2* is upregulated in EH2, while *ANT* is upregulated in PJK. Hence, this opposite expression pattern makes this *ARF2-ANT* cascade a strong candidate for future validation for investigating the molecular basis of seed size differences in these varieties.

### Identification of candidates from East European and Indian gene pools of *Brassica juncea* for improving seed quality

Utilizing the differential expression analysis between the species, we also shortlisted candidates to regulate seed coat color, oil content, and other traits (Table 1, Supplementary Table 16). One of the seed coat color genes, *TT8,* has been previously shown in *B. juncea* to exhibit perfect co-segregation with the seed coat color phenotype^70^. In our study, *TT8* ortholog B03_VARUNA_g5220.t1 showed upregulation in the brown-seeded variety (PJK) compared to the yellow-seeded variety (EH2), conforming to the expected pattern. In *B. napus*, *TT8* has also been demonstrated to block phenylpropanoid synthesis, contributing to yellow seed color^71^. Similarly, orthologs of *BAN (BANYULS)*, a late biosynthetic gene for flavonoid synthesis, were upregulated in all stages of PJK as compared to EH2. Downregulation of flavonoid biosynthesis-related genes like *BAN, TT8, TT4, TT5,* etc., have also been reported in yellow-seeded *B. napus*^25^. Moreover, various candidates related to TAG biosynthesis and metabolism were differentially expressed between EH2 and PJK. For instance, all four of the pivotal regulators of seed maturation and seed filling, *LEC1, ABI3, FUS3,* and *LEC2 (LAFL)* genes, were present among the differentially expressed transcripts in the PJK vs. EH2 comparison, and some were upregulated in EH2, while some were upregulated in PJK. These transcripts are suitable candidates for enhancing oil quality and content by contributing favorable alleles from both the varieties. Furthermore, upregulation of oil biosynthesis candidates like *DGAT1, DGAT2, FAD2, LEC2* in PJK suggests that PJK can serve as a source of trait-enhancing alleles for not just seed size, but also for oil content. Previous studies have well-established that Indian gene pools have been selected for bigger seeds and higher oil content^14^. Hence, detailed transcriptional dynamics reported here can be further used to validate candidates for marker-assisted breeding for improving both oil content and seed size simultaneously using PJK as one of the parental lines.

### Conclusions and future perspectives

We identified at least 136 cell cycle and cell division related transcripts like cyclins, *CDCs*, ^14^*Cyclophilins*, *APC* genes, *CDKs*, and other miscellaneous cell cycle-related genes that exhibit a decrease in the expression in later stages of seed development in the low seed weight variety EH2, but not in the high seed weight variety PJK. Further, we identified the candidate TFs (E2F, MYB, B3, etc.) and phytohormone-related transcripts (112 transcripts), which might act as upstream regulators of these cell cycle-related transcripts. Furthermore, eight previously known seed size-related genes (*BRI1, DET2, EOD3 MINI3, CKX2, WRI1, ANT,* and *MKK5)* were identified as the known candidates for explaining the variety-specific seed size differences. We also identified seed coat color and oil content-related transcripts differentially expressed between EH2 and PJK that can be utilized to improve oil quality. EH2 and PJK belong to distinct Indian and East European gene pools, respectively^2^. These gene pools are heterotic, and therefore have significant application in hybrid breeding in *B. juncea*^72^. The QTLs for seed size, coat color, oil content, and other seed traits have been previously reported in *B. juncea*^2,11,28,73^. However, marker-assisted breeding for improving these traits still lacks behind due to the scarcity of tightly linked markers and precise candidates. Hence, the candidates identified here (Table 1, Supplementary Tables 12 and 16) shall help address this lacuna. They can be prioritized for further validation and in crop improvement programs through genome editing or marker-assisted breeding to improve seed size and other seed-related economically important agronomic characters.

## Materials and methods

### Harvesting of samples and phenotypic analysis

Two varieties of *B. juncea*, including a bold-seeded variety Pusajaikisan of Indian origin and a small-seed weight East European variety EH2, were used for RNA sequencing. Plants were grown from October to March 2018 under open field conditions. Hand pollinations were performed as described previously^74^. Seeds were harvested at three developmental stages, i.e., 15, 30, and 45 days after pollination (D), immediately frozen in liquid nitrogen and stored at − 80 °C for RNA isolation. Seed weight was measured using five biological replicates of 20 seed each, and the average seed weight was calculated in mg. Thousand seed weight of mature seeds was calculated as described earlier^2^.

### RNA isolation and sequencing

Total RNAs were isolated using the Spectrum ™ Plant Total RNA kit (Sigma-Aldrich) as per manufacturer’s guidelines. Genomic DNA contamination was removed from the samples using the Turbo DNase kit (Ambion) following the manufacturer’s instructions. RNA quality was checked using nanodrop, agarose gel electrophoresis, and Bioanalyzer. A total of 18 seeds samples with RIN > 7 were selected to prepare libraries using the Illumina Truseq™ RNA Sample Prep kit as per the manufacturer’s instructions. Paired-end sequencing was performed using Illumina HiSeq 2500 with an average read length of 100 bp.

### Analysis of RNA sequencing data

Quality filtering at Q30 and adaptor trimming were carried out using FastP^75^. High-quality reads were mapped to the genome assembly of *B. juncea* variety Varuna^31^ using HISAT2^76^. Further, the aligned reads were assembled using StringTie v2.1.7^77^ and expression was estimated as counts using featureCounts v.2.2.0 with default parameters^78^. The gene expression level was quantified in TPM^79^ (transcripts per million). The correlation coefficient among biological replicates was calculated using Spearman’s correlation coefficient using the R package. Pairwise differential expression analysis was performed between different developmental stages/ varieties using the R package DESeq2 v.1.32.0^78^. The significance of differential gene expression was considered by the cut-off threshold of absolute log2 fold change ≥ 1 (upregulated transcripts) or ≤ − 1 (downregulated transcripts) and Benjamini- Hochberg adjusted *P*-values < 0.05 (false discovery rate).

### Functional analyses of transcriptomic data

*Arabidopsis* orthologs of *Brassica* transcripts were identified using BLASTx with TAIR protein (https://www.arabidopsis.org/download/index-auto.jsp?dir=/download_files/Proteins) sequences at an e-value of e < 10^–3^. Pathway mapping and enrichment analysis were carried out using MapMan (https://mapman.gabipd.org/mapman) as described earlier^80^. A *p*-value cut-off of  ≤ 0.05 was used for enrichment analysis, followed by Benjamini Hochberg correction with a q-value cut-off of ≤ 0.05. Gene ontology analysis was performed using AgriGO (http://systemsbiology.cau.edu.cn/agriGOv2/c_SEA.php). GO enrichment analysis was performed using a p-value cut of ≤ 0.05 followed by Hypergeometric test with the Bonferroni analysis method. Transcription factors were downloaded from Plant Transcription Factor Database (PTFDB v5.0) (http://planttfdb.gao-lab.org/). Heatmaps were generated using the MeV tool^81^. For identification of expressed transcripts, k-means clustering was performed using MeV. K-means clustering was performed to obtain 8 clusters using Pearson correlation distance metric with maximum number of iterations set to 1000. To identify candidate genes for seed-related traits, the functional_descriptions related to “seed” traits were downloaded from TAIR (https://www.arabidopsis.org/index.jsp) database. ARALIP (http://aralip.plantbiology.msu.edu/pathways/pathways) was used to identify triacylglycerol synthesis-related genes. For extracting glucosinolate biosynthesis-related genes, BRAD (http://brassicadb.cn/#/) database was used.

The use of plants in the present study complies with international, national, and/or institutional guidelines.

## Supplementary Information

Supplementary Information.

## Data Availability

The transcriptome datasets generated in this study are available in NCBI Sequence Read Archive (SRA) for the Bioproject: PRJNA824648 (https://dataview.ncbi.nlm.nih.gov/object/PRJNA824648?reviewer=c9bo4j34sniee91l288jg4eh73). Additional datasets supporting this study are included in the paper and in the supplementary files.
